# Full transcription of the chloroplast genome in photosynthetic eukaryotes

**DOI:** 10.1038/srep30135

**Published:** 2016-07-26

**Authors:** Chao Shi, Shuo Wang, En-Hua Xia, Jian-Jun Jiang, Fan-Chun Zeng, Li-Zhi Gao

**Affiliations:** 1 Plant Germplasm and Genomics Center, Germplasm Bank of Wild Species in Southwest China, Kunming Institute of Botany, the Chinese Academy of Sciences, Kunming 650204, China; 2 University of the Chinese Academy of Sciences, Beijing 100039, China; 3 Faculty of Life Science and Technology, Kunming University of Science and Technology, Kunming 650093, China

## Abstract

Prokaryotes possess a simple genome transcription system that is different from that of eukaryotes. In chloroplasts (plastids), it is believed that the prokaryotic gene transcription features govern genome transcription. However, the polycistronic operon transcription model cannot account for all the chloroplast genome (plastome) transcription products at whole-genome level, especially regarding various RNA isoforms. By systematically analyzing transcriptomes of plastids of algae and higher plants, and cyanobacteria, we find that the entire plastome is transcribed in photosynthetic green plants, and that this pattern originated from prokaryotic cyanobacteria — ancestor of the chloroplast genomes that diverged about 1 billion years ago. We propose a multiple arrangement transcription model that multiple transcription initiations and terminations combine haphazardly to accomplish the genome transcription followed by subsequent RNA processing events, which explains the full chloroplast genome transcription phenomenon and numerous functional and/or aberrant pre-RNAs. Our findings indicate a complex prokaryotic genome regulation when processing primary transcripts.

Genome-wide transcriptions of the eukaryotes are incredibly complex^1^. Widespread bidirectional promoters generate pervasive genome transcription, and transcriptions can originate from both genic and intergenic regions that have no well-defined functional elements, resulting in substantial transcription of long (>200 bp) and short (<200 bp) RNAs^2^,^3^. The long precursor RNAs (both coding and noncoding) can be further processed into shorter RNAs^4^,^5^. Together, these processes generate an unexpected genome transcriptional output. This eukaryote transcription complexity was well studied in yeast^2^,^3^, *Drosophila*^5^, and human cells^6^,^7^, but it remains poorly understood in prokaryotes, such as plastids^8^,^9^,^10^, leading to the idea that only eukaryotes harbor complex genome transcription and procession systems.

Despite living in host eukaryotic cells for approximately 1 billion years since the endosymbiosis event, the plastid still preserves its prokaryotic characteristics^11^. Previous studies suggested some prokaryotic features of plastids (e.g., prokaryotic-type gene promoters and terminators and clustered gene transcripts)^8^,^11^. It has long been considered that some chloroplast (cp) functional genes are transcribed as polycistronic transcripts that are subsequently processed into small mature RNAs, potentially indicating limited transcriptional units within the plastome (about 20 major transcriptional units; see Supplementary Table S1 for previously identified transcription units)^12^,^13^, and many of these un-transcribed regions (e.g., regions between two transcription units; ≥40% of all genomic regions). Under such a polycistronic operon transcription model, plastome genes would be transcribed from intrinsic promoters and later form stable, size-fixed transcripts. However, this model cannot account for all the transcriptional products at whole-genome level, such as tremendous plastid noncoding RNA output^10^,^14^,^15^, pseudogene transcription^16^, multiple alternative promoters/terminators^17^,^18^, numerous heterogeneous and overlapping transcript isoforms^19^, and gene transcription uncoupling in the same polycistron^20^,^21^. These transcriptional dynamics and heterogeneity suggest that an additional general transcriptional mechanism triggers whole plastome transcription.

## Results and Discussion

### The entire plastome is transcribed in higher plants and algae

In plastids and bacteria, polyadenylation of the precursor transcripts serves as a necessary process for precise cleavage of functional RNAs and rapid degradation of non-functional RNAs^22^,^23^. Thus, the assessment of polyA^+^ transcripts is suitable for the analyses of RNA metabolisms in plastids because it takes account of mRNA processing and transcription^24^. The total plant cell transcriptome includes both nuclear and organelle (chloroplast and mitochondrion) transcripts, while traditional transcriptome analyses only focus on nuclear transcripts. We first isolated the plastid transcriptome (p-transcriptome) data from the total transcriptomes for three higher plants, rice (*Oryza sativa*), maize (*Zea mays*), *Arabidopsis* (*Arabidopsis thaliana*), one green algae *Chlamydomonas* (*Chlamydomonas reinhardtii*), and one basally diverging unicellular glaucophytes *Cyanophora paradoxa* with recently published polyA^+^ transcriptome datasets (Supplementary Table S2). For the three higher plants, the transcriptome reads were from single tissue samples of shoots or leaves, except for *Arabidopsis*, which was from seedlings and flowers. The *Chlamydomonas* and glaucophytes reads were from cells cultured under normal conditions (Supplementary Table S2). After strict sequence quality control (See Experimental Procedures), transcriptome reads from each species, varying from 119 to 587 million, were further mapped to their own plastomes using a stringent pipeline (Supplementary Table S3).

**Figure 1:**
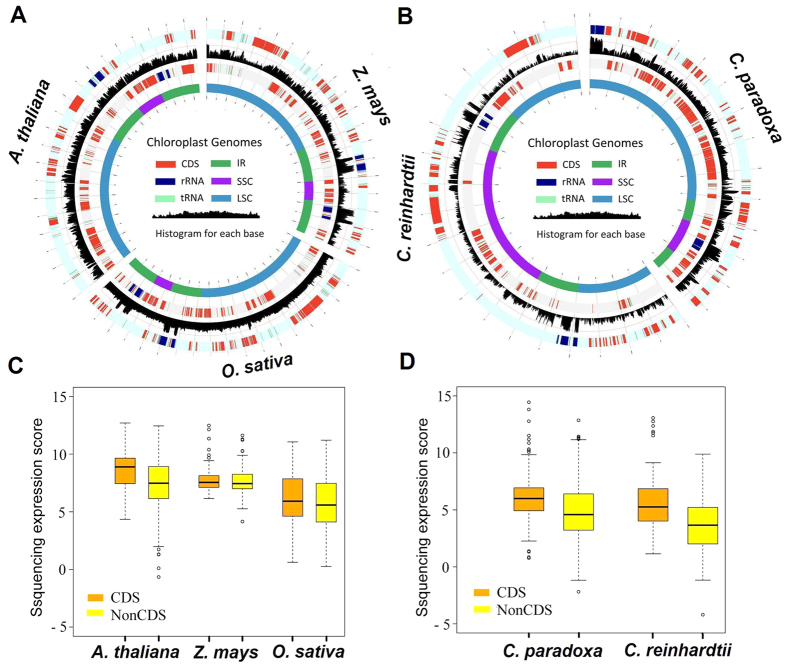
Full transcription of the photosynthetic eukaryote chloroplast genomes. (**A**,**B**) Integrated maps of the plastome transcription with the outer and third tracks representing the plastome genes, the inner track showing four genomic regions of the plastome, and the black histogram of the second track representing RNAseq reads mapping (scale log_10_-transformed numbers of sequence reads per nucleotide). The three species, rice, maize, and *Arabidopsis* were integrated into (**A**) and two unicellular algae *Chlamydomonas* and *C. paradoxa* were integrated into (**B**). The genome map was proportional to its actual genome size. The separated transcription maps for these five species are also shown in Supplementary Fig. S1. Box-and-whisker plots (in which the whiskers denote the 5th and 95th quantiles) of log_2_-transformed numbers of sequence reads per nucleotide for all intergenic sequences (NonCDS) and coding sequences (CDS). Diamonds represent outliers.

Interestingly, we found that the complete plastomes were covered by transcriptome reads (>99% for each species) with considerable read depths (from 480 to 47,875, depending on the total data; Fig. 1, Supplementary Fig. S1 and Supplementary Table S3). The transcriptome sequence reads may represent processed primary transcripts that are produced from precursor transcripts, with nearly full coverage of cp transcriptome reads mapped to the plastome, indicating the basal transcription nature of the entire plastomes of plants and algae. In *Chlamydomonas*, the initial genome coverage (about 91%) was relatively low. The *Chlamydomonas* plastome contains more than 20% repetitive sequences^25^, and this may result in reduced coverage (only one location was allowed for reads mapping, see Experimental Procedures). Indeed, after removing the repeat sequences of the *Chlamydomonas* plastome, the coverage exceeded 99%. For all the examined species, intergenic regions were also hit by substantial sequence reads, only slightly lower than that for coding regions (Fig. 1C,D), further suggesting that the intergenic regions are highly transcribed and that the removal of intergenic regions is not necessary for the polyadenylation/degradation of plastid primary transcripts. Reads mapping resulted in a few unmapped regions (~1% of the total genome), of which >90% had a sequence length <30 bp. We then validated the entire plastome transcription in rice by using reverse transcription polymerase chain reaction (RT-PCR) to confirm that all genomic regions we examined were indeed transcribed (Supplementary Table S4). Collectively, our transcriptome analyses provide direct evidence for whole-genome transcription in both green plants and algae.

To examine tissue-specific transcription of the entire plastome, we analyzed rice genome transcription profiling for seven different tissues (callus, leaf, panicle before and after flowering, root, seed, and shoot; Supplementary Table S5). To reduce the influence of rRNA and unequal sequence reads, we deleted rRNA sequence reads and normalized the datasets from all tissues to have the same number of sequence reads (~38.9 million) selected at random. The results of reads mapping to the rice plastome showed that the coverage of transcribed regions varied from 35% in root to 75% in leaf (Supplementary Table S5). In addition, we generated and analyzed rice transcriptome datasets (~52.6 million for each tissue) with in-depth sequencing for four tissues (leaf, panicle before flowering, root, and shoot). The reads mapping results revealed elevated coverages of transcribed regions that varied from 73% in root to 99% in shoot (Supplementary Table S5). Taken together, the analyses of two datasets with different sequencing depths suggest that the tissues with greater photosynthetic activity such as the leaf and shoot exhibit higher levels of chloroplast transcripts.

**Figure 2:**
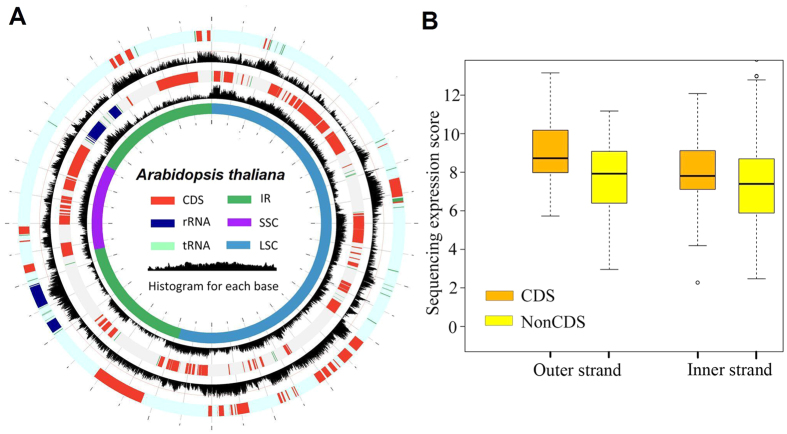
Both strands of the *Arabidopsis* plastome were transcribed. (**A**) Strand-specific transcriptome reads were mapped to both strands of the *Arabidopsis* plastome. The outer and third tracks represent genes in the outer and inner strand, respectively. The black histograms of the second and fourth tracks indicate RNAseq reads mapping (scale log_10_-transformed numbers of sequence reads per nucleotide). (**B**) Comparisons of intergenic and coding region transcription for each strand of the *Arabidopsis* plastome. Box-and-whisker plots (in which the whiskers denote the 5th and 95th quantiles) of log_2_-transformed numbers of sequence reads per nucleotide for all intergenic sequences (NonCDS) and coding sequences (CDS). Diamonds represent outliers.

**Figure 3:**
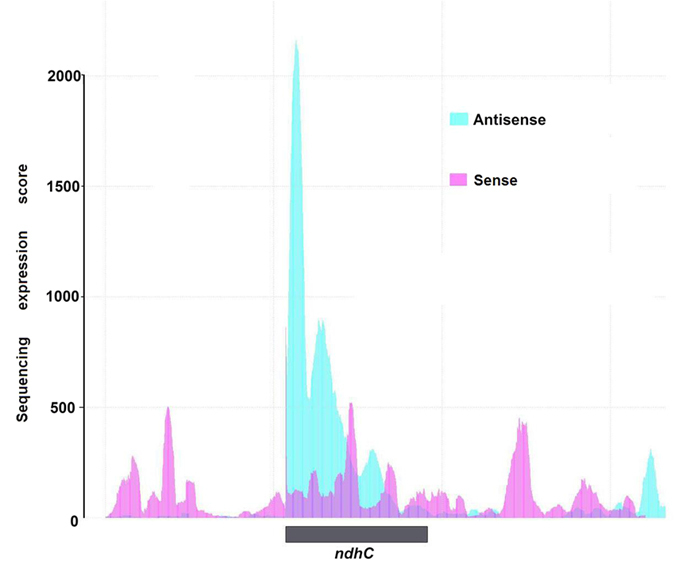
Antisense transcription in the chloroplast genome. Strand-specific transcriptome reads showing that antisense transcription (light blue) exceeds sense transcription (light red) for the *ndhC* gene.

We also aligned ~133 million strand-specific RNA-sequencing (RNA-Seq) reads of *A. thaliana* to its plastome (Supplementary Table S2). Although the numbers of mapped reads were low compared with non-strand specific transcriptome reads (~224.8 million) (Table S2), >94% was covered for each strand (Fig. 2A and Supplementary Table S3). While calculating the read distribution for each strand, we found that both the coding and non-coding regions were almost equally covered by all mapped reads (Fig. 2B). These findings demonstrate that antisense transcription occurs for both strands of the entire plastome and is most likely associated with long non-coding RNAs (lncRNAs) transcription (Figs 2A and 3)^26^.

### Exclusion of nuclear-localized plastid DNAs (nupDNAs) transcription

**Figure 4:**
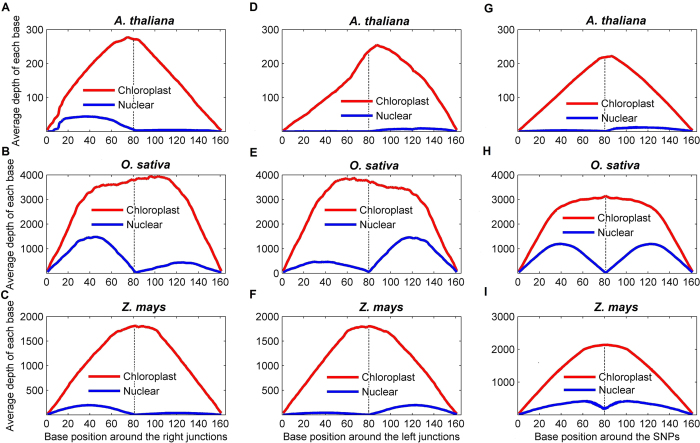
Examination of nupDNA transcription. (**A**–**F**) nupDNAs (blue) and plastome sequences (red) with a homologous sequence before (**A**–**C**) and after the site 80 (**D**–**F**). (**G**–**I**) nupDNAs (blue) and plastome homologous sequences (red) with a single SNP or indel in site 80. All nupDNAs and plastome homologous sequences had a sequence length of 160 bp (x-axis). The y-axis represents the RNAseq reads mapping depth per nucleotide.

NupDNA fragments were thought to be quite common in plant nuclear genomes, and they should be non-functional, indicating that they would be rapidly fragmented and eliminated from the nuclear genome during evolution^27^,^28^. The transcriptome data in the present study were generated from whole-cell preparations, providing the possibility that some transcriptome reads may come from the nupDNA transcripts. We counted the reads depth at the positions that were variable between nupDNAs and the chloroplast reference genome. The reads depths of the regions that contain variable positions or junctions were significantly lower and close to zero compared to those covering non-variable positions and the corresponding chloroplast genomic regions (Fig. 4), indicating that the nupDNAs were generally not transcribed or transcribed at comparatively low levels. Besides, a plant cell often harbors hundreds of chloroplast genomes (400 to 1,600 chloroplast genome in a leaf cell)^29^, therefore, when sequence reads of hundreds of high quality chloroplasts are aligned, the nupDNA transcripts, if present, can be neglected.

Moreover, the above-mentioned rice tissue-specific reads mapping results showed that after sequence reads normalization and rRNA depletion, the mapped plastid transcriptome reads were 0.02%, 0.06%, 2.28%, and 2.61% in root, callus, leaf, and shoot, respectively (Supplementary Table S5), which is consistent with increased photosynthesis abilities in plastids. Among the studied species, the rice genome exhibited the largest proportion of nupDNAs^27^,^29^. However, both nupDNA and tissue-specific transcription patterns indicate that the p-transcriptome reads mapping results reflect the actual plastome transcription.

### The entire genome transcription of cyanobacteria

**Figure 5:**
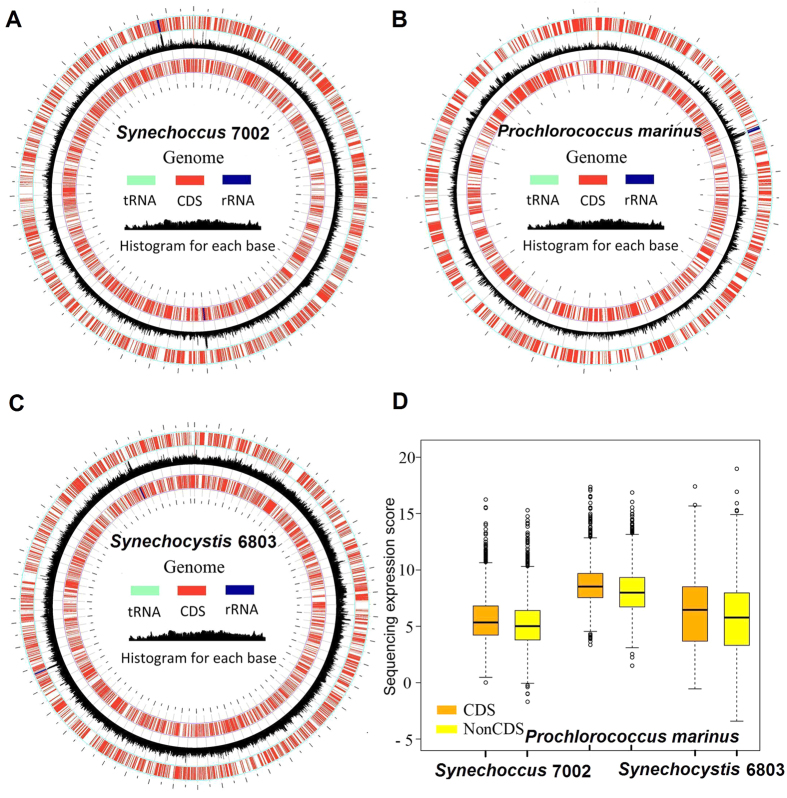
Full transcription of the cyanobacteria genomes. (**A**–**C**) Maps of the cyanobacteria genomes transcription with the outer and third tracks representing genes in the genome, and the black histogram of the second track represent RNAseq reads mapping (scale log_10_-transformed numbers of sequence reads per nucleotide). (**D**) Comparisons of intergenic and coding region transcription for the five species. Box-and-whisker plots (in which the whiskers denote the 5th and 95th quantiles) of log_2_-transformed numbers of sequence reads per nucleotide are shown for all the intergenic sequences (NonCDS) and coding sequences (CDS). Diamonds represent outliers.

Cyanobacteria are prokaryotes thought to be related to the evolutionary ancestors of the chloroplasts^8^,^11^. To investigate whether full transcription of the algae and plant plastomes was derived from cyanobacteria^30^, we analyzed three cyanobacteria with high-quality reference genomes and high-throughput transcriptome datasets: *Synechocystis* sp. PCC 6803, *Synechococcus* sp. PCC 7002, and *Prochlorococcus marinus* subsp. *pastoris* str. CCMP1986. Even though their genome sizes varied from 1.6 to 3.5 Mbp, the transcriptome reads mapping showed that they were almost entirely transcribed (at least 94%) (Fig. 5 and Supplementary Table S3). These reads were nearly evenly mapped to both coding and non-coding regions (Fig. 5D). Thus, cyanobacteria genomes may share the same transcription mechanism with plant plastomes, indicating a common ancestral origin of transcription.

### RNA editing

Plastome transcripts undergo RNA editing that change specific cytosines (Cs) in organelle mRNAs to uracils (Us) in the land plants^8^. Chloroplast RNA editing was hypothesized to have evolved simultaneously with the origin of the first land plants^31^ because it was poorly observed in plastid-encoded RNAs of algae groups^8^. The high-throughput RNA-Seq data allow the generation of a comprehensive view of RNA editing at whole-genome level. By further examining the reads mapping results of the transcriptomes, we detected 91, 208, and 51 RNA editing sites in the rice, maize, and *Arabidopsis* plastomes, respectively (Supplementary Table S6). Moreover, 69 and 75 editing sites were found in *Chlamydomonas* and *C. paradoxa*, respectively (Supplementary Table S6). Interestingly, only 6, 15, and 43 editing sites were observed in *P. marinus*, *Synechococcus*, and *Synechocystis*, respectively (Supplementary Table S6). Some genes involved in photosynthetic metabolism (e.g., *psa*-, *psb*-, *pet*-, *atp*-, and *ndh*-genes) or gene expression system (e.g., *rpl*-, *rps*-, and *rpo*-genes) were also frequently edited in the cyanobacteria genomes. While, conserved editing sites within these genes from these examined species were quite spare, this may be partially owing to frequent gene sequence variation among them. Thus, our results support the hypothesis that RNA editing emergence preceded chloroplast endosymbiosis^32^.

### *De novo* plastome assembly from transcriptome data

**Figure 6:**
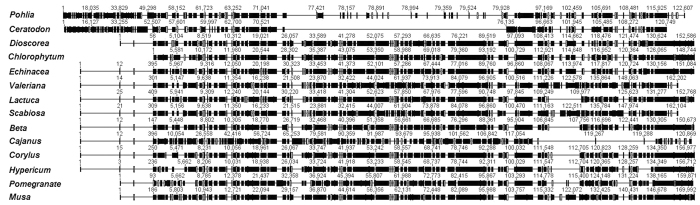
Complete cp genomes were *de novo* assembled from transcriptome data. The wrap sequence alignment of the assembled genome. The black blocks depict genome similarity for these species. A detailed species list is provided in Supplementary Table S7.

The evidence for whole-genome transcription suggests that the entire genome can be transcribed into RNAs. Conversely, this finding implies that the plastome sequence can be straightforwardly assembled from the transcriptome. To test this, we sampled a total of 14 plant transcriptome datasets downloaded from NCBI Transcriptome Shotgun Assembly (TSA) database. The complete plastomes were *de novo* assembled from these species, which included 2 bryophytes and 12 angiosperms (Fig. 6, Supplementary Table S7). This added an extra layer of evidence for whole-plastome transcription in photosynthetic eukaryote chloroplasts.

### A multiple arrangement transcription model

It has long been thought that some plastome genes were transcribed via typical polycistronic operon transcriptional model as observed in *Escherichia coli*^8^,^11^. Recently, a novel genome-wide transcriptional start site (TSS) category assignment was reported in both chloroplast and cyanobacterial genomes^14^,^33^,^34^, which identified numerous promoters inside open reading frames (ORFs), non-coding regions, antisense to known genes, and genomic regions without any predicted genes. These functional TSSs far exceeded the numbers of genes within gene clusters^14^,^33^,^34^. Furthermore, the promoter-like sequences, including “-10,” “-35,” and YRTa motifs, are quite divergent between different plastomes and genes within the same genome^17^. Moreover, inefficient transcription termination is a well-established characteristic of plastid gene expression, and many transcripts possess variable 3′ extensions^19^.

**Figure 7:**
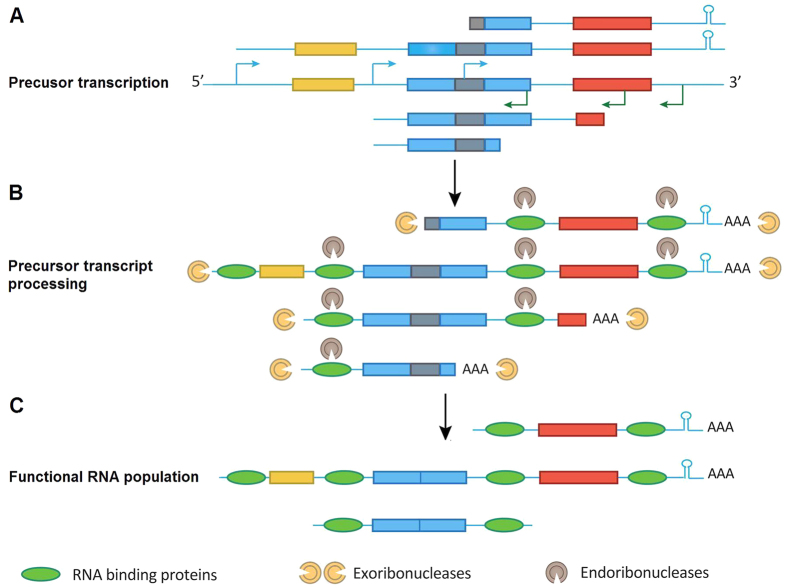
Model for the full plastome transcription and procession. (**A**) Transcription initiation of a gene cluster occurs from multiple promoters (bent arrow) upstream of open reading frames (ORFs) or within ORFs. Together with inefficient transcription termination, this setup generates numerous precursor transcripts that can include complete or incomplete ORFs. Introns and RNA stem–loop structures are depicted as light black rectangles and hairpins, respectively. (**B**) Precursor transcripts are processed by a combination of exo- and endo-ribonucleases. The precursor transcripts also can be polyadenylated by the addition of a Poly(A)-tail at the 3′-end of the transcripts. The sequence-specific RNA-binding proteins define functional RNAs followed by ribonuclease digestion. Introns and incomplete ORFs without sequence-specific RNA-binding proteins protection were digested by exo- or endo-ribonucleases. (**C**) RNA processing produces a pool of functional RNAs.

**Figure 8:**
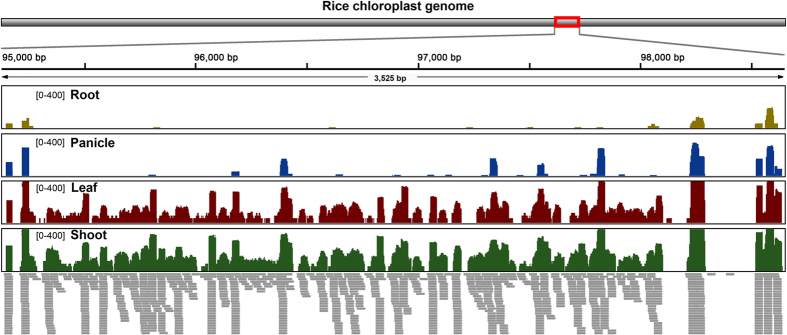
Small RNA transcription in the rice chloroplast genome. Small RNA transcriptome reads of four tissues were mapped to the rice plastome. The colored histograms represent small RNA mapping coverage in a logarithmic scale. Detailed statistics of reads mapping is given in Supplementary Table S8.

Considering the extensive transcription initiation and infrequent and stochastic termination described above and the observed full transcription of the plastomes (Fig. 1), we propose a multiple arrangement transcription model for the entire transcription of plastomes (Fig. 7). Briefly, plastome transcription can initiate the upstream of a gene and/or internal to a gene, using TSSs as described previously^14^,^33^,^34^, and inefficient transcription termination creates many precursor transcripts with variable 3’ ends (Fig. 7)^19^. This generates numerous overlapping precursor transcripts with variable sizes that cover both strands of the entire genome. Because the precursor transcripts are likely to be transcribed from various combinations of start and termination sites, many transcripts can include incomplete ORFs and pseudogenes^16^. These primary RNAs are finally processed and spliced by many nucleus-encoded chloroplast ribonucleases to form mature RNAs (mRNAs and small RNAs) (Fig. 7)^15^,^35^. Reads mapping of small RNA sequences showed that substantial small RNAs covered the entire plastome (Fig. 8 and Supplementary Table S8).

The model presented here can feasibly explain the large RNA transcription outputs in algae and plant plastomes, possibly also in cyanobacteria. Previous studies have genome-wide identified numerous transcriptional start sites (TSS) in both chloroplast (e.g., barley)^14^ and cyanobacterial genomes^33^,^34^. Thus, the mechanism of plastome transcription proposed in such a model may not be confined by intrinsic gene transcriptional initiation and termination. Multiple transcription initiation and termination form the basis for full transcription of the plastomes. This transcription can start and stop from several genomic locations, generating numerous long and short transcripts that can overlap. The process may reflect non-specific combinations of a series of sigma factors and RNA polymerases to the DNA for transcription initiation and termination. After transcription, the long precursor RNAs (both functional and non-functional) can be further processed into shorter RNAs^4^,^10^. The transcriptional diversity of RNAs together with further posttranscriptional processes generates uncountable plastome transcripts^15^. Furthermore, we observed full plastome transcription with RNA editing in cyanobacteria, indicating an ancient origin of full plastome transcription in photosynthetic eukaryote chloroplasts about 1 billion years ago.

The plastome codes functional plastid RNA polymerase (PEP) that is homologous to the cyanobacterial RNA polymerase^14^. The second polymerase, denoted as nuclear-encoded plastid RNA polymerase (NEP), which was reported to participate in plastid transcription of higher plants but not found in algae and cyanobacteria^14^. The finding that both cyanobacteria and green algae cp genomes of land plants can be fully transcribed suggests that there are not any differences regarding the transcription of the PEP and NEP-dependent transcripts among these studied species. This result is consistent to a former study on transcription initiation in barley chloroplasts that detected many transcription start sites in the genome but failed to exhibit any differences between the PEP and NEP-dependent transcripts^14^. However, it still remains largely unknown about how the RNA polymerase influences the plastid transcription.

Full plastome transcription may constitute a new level of prokaryotic genome transcriptional regulation at the level of processing of primary transcripts. One question that has emerged is why these genomes produce so many transcripts. Because many of the transcripts start/terminate from/in genic regions, these aberrant transcripts may be non-functional. We would argue that the transcription mechanism may produce many transcripts, and a post-transcriptional regulation system and external nature selection pressures will act on them to determine which transcripts should be retained. This prediction potentially indicates that external environment changes may influence genome transcription and post-transcriptional regulation. Even so, the question holds true that, based on the collected data, we still cannot assess how many transcripts are transcribed according to the “multiple arrangement transcription model”. Further studies are still needed to examine that to what extent plastome transcripts are governed by this model.

## Methods

### Transcriptome data

Transcriptome reads of three higher plants (rice, maize, and *Arabidopsis*), two unicellular algae (*Chlamydomonas* and *C. paradoxa*), and three cyanobacteria were downloaded from the National Center for Biotechnology Information (NCBI) Short Read Archive database (http://www.ncbi.nlm.nih.gov/sra/). Considering high sequence quality and sufficient depths, we selected transcriptome data that were generated and released by different laboratories. The accession numbers for each species are described in Supplementary Table S2. The rice, maize, *Arabidopsis*, *Chlamydomonas*, and *C. paradoxa* plastomes, as well as the three cyanobacteria genome annotation files (GenBank format) were downloaded from NCBI (http://www.ncbi.nlm.nih.gov/nuccore/).

### Data processing and reads mapping

Raw reads in FASTQ format were trimmed with the SolexaQA package^36^ to remove adapters and low quality bases (parameters: -h 20 –b, -l 30). The filtered RNA-seq reads (Phred quality scores >20, length >30) were then mapped to the responding plastome using Bowtie (parameters: –best, -S, default options otherwise)^37^. The following stringent alignment parameters were applied to properly align reads to the chloroplast genome: 1) reads that aligned to multiple genomic locations were ignored; and 2) of the uniquely mapped reads, tolerances were set to allow at most one mismatch. Then, the SAMtools package was employed to index the alignment results as BAM files. The coverage and base depth were calculated by converting the BAM alignments into pileup files that were used for further statistical analyses of plastome transcription.

### Calculation of plastome transcription

Based on the plastome annotation files, we calculated transcription in the coding and non-coding regions of the plastomes. The position information for all coding regions (protein-coding, rRNA, and tRNA genes), and non-coding regions (intergenic regions and introns) were extracted from the annotation file with perl scripts. The transcription level for every genomic base pair position was assigned on the basis of how many sequence reads covered each position. The log_10_ of the score for each base pair position was plotted with Circos (Figs 1A,B and 5A–C, Supplementary Fig. S1)^38^. The log_2_ of the score for each base pair position of all intergenic sequences (NonCDS) and coding sequences (CDS) (Figs 1C,D and 5D) were plotted with R/Bioconductor.

### Examination of nupDNAs transcription

Since a large number of chloroplast-derived sequences exist in the nuclear genome (nupDNAs), we performed an additional analysis to ensure that cp-transcriptome reads did not contain RNA transcripts from nupDNAs. We first searched for nupDNAs in the nuclear genomes of rice, maize, and *Arabidopsis* by using their plastome sequences as BLAST queries and E-values of <10^−10 (^27,29). The genome sequence of *Arabidopsis* (*Arabidopsis thaliana*, version 9.0) was downloaded from The *Arabidopsis* Information Resource (http://www.arabidopsis.org/). O. *sativa* genome sequences (version 7.0) maintained by Michigan State University (http://rice.plantbiology.msu.edu/) were used for rice. Maize (*Z. mays*) genome sequence (release 4a.53) was downloaded from http://www.maizesequence.org/. A BLAST search identified thousands of nupDNAs with high homology to the plastome sequences. Considering only large nupDNAs fragments could be transcribed in the nuclear genome, we filtered the nupDNA fragments ≥500 bp with ≥95% similarity for further analyses. We kept 160-bp regions within these sequences that matched the chloroplast genome in line with the mapping strategy of nupDNAs that did not allow any mismatch for reads mapping. To discriminate authentic plastome transcriptions during reads mapping, we identified positions that were variable between nupDNAs and the chloroplast reference sequence and calculated reads depth for the following nupDNAs: 1) nupDNAs sequence (160 bp) containing a nuclear genome sequence of 80 bp in one side (left or right) with the middle site (site 80) serving as a junction; and 2) the nupDNA sequences harboring a single insertion/deletion (indel) or single-nucleotide polymorphism (SNP) differences with plastid DNA in the site 80 and with a total sequence length of 160 bp (Fig. 4). Because we did not allow any mismatch during reads mapping, we expected that the reads depth would decline in the junction of the nupDNAs and in sites with indel or SNP differences.

### RNA editing sites

To identify RNA editing sites, all the transcriptome reads were again mapped to the plastome using PASS software (version 1.62)^39^. The uniquely mapped reads with size ≥30 bp and Phred quality scores >20 were reserved (parameters: -flc 1, -fid 90, -fle 30, -gff, -info gff, -trim 5 20). The reads mapping results (GFF file) were then used to identify C-to-U changes and other editing events due to RNA editing in the plastome by the PASS_SNP program (parameters: -f 0.5 -q 20 -c 10 2000). Briefly, the PASS_SNP program took the alignment file (GFF file) as input and identified putative RNA editing sites, checking quality, coverage, and frequency for each base transition. A site was considered potentially edited if reads depth ≥10 and 5 or more Us are in the aligned reads at the same position. Nucleotides with a 100% change rate between RNA and the genome sequence were considered SNPs^40^.

### Plastome assembly

To assess the power of plastome assembly from transcriptome data, we downloaded 14 sets of transcriptome data from the NCBI Short Read Archive (Supplementary Table S7). The species with transcriptome data ≥4 Gb and no plastome reported up to June 2012 were selected for study. Transcriptome reads were first filtered by BLAST to all the sequenced plastome sequences and then *de novo* assembled using SOAPdenovo^41^ as previously described^16^.

### Transcriptome sequencing

To further examine plastome transcription in rice, we used *O. sativa* ssp. tropical *japonica* (IRRI Accession No. 24225) for transcriptome sequencing. Four organs from different developmental stages were collected from this strain of rice, including root and shoot at the 30-d seedling stage, flag leaves at the tillering stage, and panicle at the booting stage. Total RNA was extracted using a standard phenol/chloroform RNA isolation method, followed by treatment with DNase I for 30 min at 37 °C to remove residual DNA. For high-throughput sequencing, the sequencing library was constructed by following the manufacturer’s instructions (Illumina) for paired-end 100 bp × 2 sequencing. Sequence reads mapping was the same as the other transcriptome data.

### Small RNA sequencing

Apart from transcriptome sequencing, total RNA from the same four rice tissues were used to construct small RNA libraries and then sequenced with Solexa sequencing technology (Illumina). Both the transcriptome and small RNA sequences were aligned to the rice plastome using the reads mapping strategy described above.

### Experimental validation of plastome transcription using RT–PCR

Total RNA was extracted from leaves of rice and then dissolved in nuclease-free water and treated with DNase I for 30 min at 37 °C to remove possible DNA contamination. For RT-PCR, we designed PCR primers that covered the entire rice plastome except the second inverted repeat region. The primers for each element are listed in Supplementary Table S4. RT-PCR was conducted using the following reagent in a 30-μl PCR reaction volume: 3 μl cDNA, 3 μl 10× Thermo Buffer, 0.6 μl primer 1, 0.6 μl primer 2, 0.6 μl dNTPs (10 mM), 21.9 μl ddH_2_O, 0.3 μl Taq-Polymerase. The following temperature cycle was used: initial denaturation at 94 °C for 5 min, followed by 30 cycles of denaturation at 94 °C for 30 s, annealing at 48–54 °C according to the optimal primer requirements (for 30 s), and elongation at 72 °C for 1 min, ending with a 10-min elongation step at 72 °C. PCR fragments were visualized on 1% agarose gels.

## Additional Information

**How to cite this article**: Shi, C. *et al*. Full transcription of the chloroplast genome in photosynthetic eukaryotes. *Sci. Rep.* **6**, 30135; doi: 10.1038/srep30135 (2016).

## Supplementary Material

Supplementary Information

Supplementary Tables
