# Supplementary material for: Full transcription of the chloroplast genome in photosynthetic eukaryotes
Source: Sci Rep. 2016 Jul 26;6:30135. doi: 10.1038/srep30135 (PMC4960489; doi:10.1038/srep30135)
Supplement: Supplementary Information [file srep30135-s1.pdf]

Supplemental Information

Full transcription of the chloroplast genome in photosynthetic eukaryotes

Chao Shi[1,3*], Shuo Wang[2*], En-Hua Xia[1,3*], Jian-Jun Jiang[2], Fan-Chun Zeng[2], and Li-Zhi Gao[1, 2 **]

[1]Plant Germplasm and Genomics Center, Germplasm Bank of Wild Species in Southwest China, Kunming Institute of Botany, the Chinese Academy of Sciences, Kunming 650204, China

[2]Faculty of Life Science and Technology, Kunming University of Science and Technology, Kunming 650093, China

[3]University of the Chinese Academy of Sciences, Beijing 100039, China

*These authors contribute equally to this work.

**To whom correspondence should be addressed. Tel./Fax: +86-871-5223277, Email: Lgao@mail.kib.ac.cn

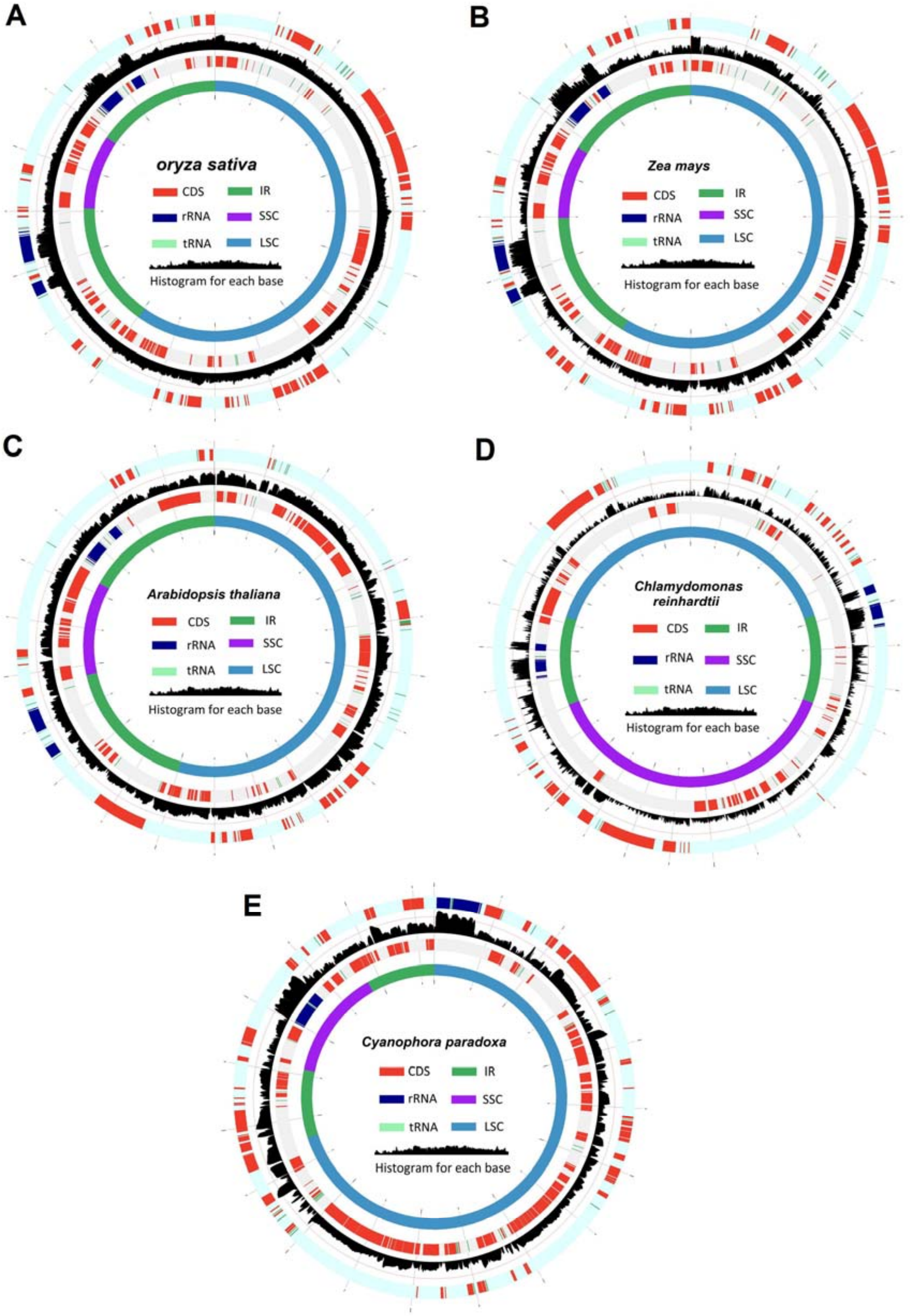

Supplementary Figure 1. Full transcription of the photosynthetic eukaryote chloroplast genome. This is a enlarged version of the map in **Figure 1A and B.** The outer and third tracks represent the plastome genes, the inner track shows the four

genomic regions of the plastome, and the black histogram of the second track represents RNAseq reads mapping (scale log_{10}-transformed numbers of sequence reads per nucleotide).

Supplementary Table 1. The representative transcription units of chloroplast genes in higher plants.

Representative transcript units in higher plants*	
1	*atpB-atpE*
2	3’ *rps12-rps7*
3	*psbE-psbF-psbL-psbJ*
4	*psbD-psbC-orf62* (in dicots)
5	*psbK-psbI-psbD-psbC-orf62-trnG* (in monocots)
6	*ndhC-ndhK-ndhJ*
7	16S *rDNA-trnl-trnA*-23S *rDNA*-4.5S *rDNA*-5S *rDNA*
8	*rpoB-rpoC1-rpoC2*
9	*rpl23-rpl2-rps19-rpl22-rps3-rpl16-rpl14-rps8-infA-rpl36-rps11-rpoA*
10	*trnE-trnY-trnD*
11	*clpP-rps12-rpl20*
12	*orf31-petG-psaJ-rpl33-rpsl8*
13	*psaA-psaB-rps14*
14	*psaC-ndhD*
15	*psbB-psbH-petB-petD*
16	*psbK-psbl-trnG*
17	*ndhA-ndhI-ndhG-ndhE-psaC*
18	*rpl32-sprA*
19	*rps2-atpI-atpH-atpF-atpA*

*Only major transcription units were listed. Please also see ref (*12*, *13*).

Supplementary Table 2. Summary of sequence reads used in this study.

**Species**	**Tissues**	**Sequence data**	**Reference**
O. sativa	seedling shoots	ERA000212	(*43*)
A. thaliana	seedlings and flowers	SRA047499	(*44*)
	immature floral tissue (directional sequencing)	SRA000286	(*45*)
Z. mays	leaves	SRA050928	Not available
C. reinhardtii	whole cell	SRA026231	Not available
C. paradoxa	whole cell	SRA047914	(*46*)
***Prochlorococcus marinus* CCMP1986**	whole cell	SRA050651	Not available
***Synechococcus* PCC7002**	whole cell	SRA044901	Not available
***Synechocystis* PCC6803**	whole cell	SRA025999	Not available

Supplementary Table 3. Statistics of RNA-seq reads mapping with the chloroplast genomes.

Species	Reads used for mapping	Average reads depth	Genome coverage (Bowtie) *	Genome coverage (PASS) *
A. thaliana	224,829,363	480	**98.76%**	**99.18%**
***A. thaliana* (F)** [b]	133,741,703	503	**93.33%**	**94.12%**
***A. thaliana* (R)** [b]		534	**94.25%**	**94.88%**
O. sativa	149,778,844	47,875	**99.91%**	**99.92%**
Z. mays	587,912,045	6,826	**98.53%**	**99.56%**
C. reinhardtii	575,401,566	988	**91.41% (99.34%）** [a]	**90.73%（99.16%）** [a]
C. paradoxa	119,434,879	589	**98.23%**	**98.92%**
***Prochlorococcus marinus* CCMP 1986**	59,735,881	43	**94.33%**	**94.34%**
***Synechococcus* PCC 7002**	195,192,665	78	**99.27%**	**99.27%**
***Synechocystis* PCC 6803**	459,631,042	758	**99.99%**	**99.98%**

Note: *All filtered reads were mapped using Bowtie or PASS.

[a] Genome coverage was calculated after trimming repeat sequences from the *C. reinhardtii* genome.

[b] Strand-specific sequencing data were mapped to forward (F) and reverse strand (R) of the chloroplast genome.

Supplementary Table 4. Primers used to examine intergenic region transcription in the rice plastome.

Primer	Primer sequence (5'-3')	Position	Region
1F	GCCGAGTACTCTACCATTGA	3902	*tRNA-Lys*
1R	CGTATTGTATTCTGCACGAGA	6567	*tRNA-Gln*
2F	GTTTGGGAAAGTCCGAAGAA	6921	*psbK*
2R	ACCATTTCCGAGCACCTTAT	8855	*psbD*
3F	TTTCTGTTCTGGCTCGGTT	11364	*psbC*
3R	CGTCTCCGCACTTAGCAGT	12832	*tRNA-Met*
4F	CGGACGCCTTAAGTATATCA	13677	*tRNA-Gly*
4R	TCGCCTGTCCTACTCTAAGA	16159	*tRNA-Tyr*
5F	GGCAGGAAGCAAGATCAAATA	16362	*tRNA-Asp*
5R	AACGTGGATGAATACTTGCTC	18158	*tRNA-Cys*
6F	GCAAGTATTCATCCACGTTTC	18160	*tRNA-Cys*
6R	CGTCAAGTATACCCGTTTCAA	20101	*rpoB*
7F	GTCATTGGGTTAAGAGAAGTTCCGATTG	24,319	*rpoc1*
7R	GATGATACTGTAATGGAATGGCGAACC	26,456	*rpoc2*
8F	GATTCGCGATTTCCTAAGA	27700	*rpoc2*
8R	AATGACAACGAGCCCTTATT	29804	*rps2*
9F	TATAGCTCTTCGGGAATGT	30062	*rps2*
9R	TTGTCCTGCAGCAGTACCTTGACCAACT	32114	*atpH*
10F	TCCCAGCCCTGATACCAAT	43344	*ycf3*
10R	GGCCATCTCTCCTCCATAATC	44430	*tRNA-Ser*
11F	GATTACTTCCGGATTTAGGTG	45323	*rps4*
11R	ATCTACATTCCTTGCCGATA	47274	*tRNA-Leu*
12F	CCAACTATTTGTCAGGCGACT	50878	*tRNA-Val*
12R	AGTAGGGGAACGGACTCGTGA	52689	*atpB*
13F	GGAGCTCCCGTGTCAATCACT	53022	*atpB*
13R	GAAGCCACCGGTATAACACCT	55211	*rbcL*
14F	CCGGTAGATAAACTAGATAGC	55,506	*rbcL*
14R	CGCCAACTAGATACTCCT	57,598	*psaI*
15F	CCTTTACCGCGCATTATTG	56638	*psaI*
15R	GATTGGATATCCCGCATAAG	58063	*ycf4*
16F	GCGGGATATCCAATCAATTAG	58068	*ycf4*
16R	GGGAACAGGGCCTATCACAA	60044	*petA*
17F	GTGCTTCAGGATCCATTACGC	60436	*petA*
17R	GAACTATGACACAATCAAACCCGAATG	61910	*psbL*
18F	TGATCCTTTCTTTCTCCTCGCTTCAT	61226	*psbJ*
18R	AGTCCCTAATTCCGAATCAAG	63345	*psbE*
19F	CCTGTGACATTTTGTACCC	64250	*tRNA-Trp*
19R	ACGAGCCTGTTTTATAGCA	65914	*rps18*

20F	TTCTTCTTCCTCTTCGGTTTC	65110	*rpl33*
20R	TGCCGTCACTGTATGGATAA	67070	*rpl20*
21F	CGCCATTCCTGAGATTAAC	68053	*ClpP*
21R	CACACACTCATATTCCAGAGATACCGAAAC	71214	*psbH*
22F	GTTTCGGTCGGTCTTATTGTA	77899	*rpl14*
22R	TGAGAAATTGACTCGGAAAG	78704	*rpl16*
23F	GGCCTGTTATCTCTATCAAGA	71283	*psbH*
24R	CCCCTCAATATTGCAAGTTT	73298	*petD*
25F	GACCTTGCGGATCTGTAACA	75671	*rps11*
26R	TGAGAAATTGACTCGGAAAG	78704	*rpl16*
27F	AGGTTTGAATCTCGCAATGG	78668	*rpl16*
28R	AGAAGAAGAGTGGGCTAAGGA	80506	*rpl22*
29F	ATAGTACTCCTACTGACTTCGGCTTTAGTG	80223	*rps3*
29R	GTAACGGCAGCAAGTGATTGA	82128	*rpl2*
30F	TCTTCTCTCCATCGGAACAA	87396	*ndhB*
30R	TTATTCGAGGAGCCCTAGAT	89080	*rps12*
31F	GGGCGTGGAAACAGATAGAA	90548	*orf71*
31R	GTTTACGGCTAGGACTACTGG	92044	*rrn16*
32F	ATGCAAAGCGAAGAACCTTAC	92212	*rrn16*
32R	GGTCTGTGAAGATGCGTTGTT	93491	*ycf68*
33F	GGGTGAGTCAGGGCCTAAGAT	96581	*rrn23*
34R	ATAGAGTCCGACCGCAACGAC	99684	*tRNA-Asn*
35F	ATGAGTCTACCGCTTACAAGA	101230	*rps15*
35R	GTCTATCCTCATGAACCGGGAAATACTATG	102016	*ndhF*
36F	GACACCCTTGTCGAAAAACTGCGTTAGT	101578	*rps15*
36R	GGGGTTGGTTACTAGTTCC	103213	*ndhF*
37F	AGCCCATATGCGACGAAGAT	103516	*ndhF*
37R	TGGGTAATGTAAATGAACCAT	106177	*ccsA*
38F	GCTCGTACACATTGAGTGCAT	108208	*psac*
38R	CGGGTGGAATTTGTCTATT	110875	*ndhI*
39F	TGCCACAGCTACAAAGTAGGA	109576	*ndhE*
39R	ATTGGTCTTCTTATGGCAGGATATAGCTCA	112255	*ndhA*
40F	GAGCTATATCCTGCCATAAGA	112256	*ndhA*
40R	GCGGTAATTTCATCGTTC	114018	*rps15*

Supplementary Table 5. Tissue-specific transcriptome reads mapping in the rice plastome.

A. Transcriptome reads from seven different tissues.

**Tissues**	**Normalized reads***	**Mapped reads**	**Coverage (%)**
**Root**	38,903,554	7,780 (0.02%)	32.1
**Callus**	38,903,554	23,342 (0.06%)	35.1
**Seed**	38,903,554	151,723 (0.39%)	73.1
**PAAF**	38,903,554	482,404 (1.24%)	77.1
**PABF**	38,903,554	567,991 (1.46%)	76.8
**Leaf**	38,903,554	887,001 (2.28%)	73.6
**Shoot**	38,903,554	1,015,383 (2.61%)	78.4

B. Transcriptome reads from four different tissues and with higher in-depth sequencing.

**Tissues**	**Normalized reads***	**Mapped reads**	**Coverage (%)**
**Root**	52,627,968	63,154 (0.12%)	72.6
**PABF**	52,627,968	931,515 (1.77%)	89.4
**Leaf**	52,627,968	1,405,166 (2.67%)	99.3
**Shoot**	52,627,968	1,505,159 (2.86%)	99.7

*All reads were normalized to contain same number of reads by randomly extraction from different tissues.

Supplementary Table 7. *De novo* chloroplast genome assembly from transcriptome data.

Taxa	Species	Data	Plastome assemble length
Bryophytes	*Pohlia nutans*	SRP012293	122,067
	Ceratodon purpureus	SRX032867	120,749
angiosperms	*Dioscorea villosa*	SRX060329	152,589
	Chlorophytum rhizopendulum	SRX116253	148,744
	Echinacea purpurea	SRX085116	151,084
	Lactuca serriola	SRX098217	162,202
	Valeriana officinalis	SRX047631	152,768
	Scabiosa columbaria	SRX090087	162,104
	Beta vulgaris	ERX026027	150,673
	Cajanus cajan	SRA030523	120,869
	Corylus avellana	SRX154512	156,977
	Hypericum perforatum	SRX062064	156,712
	Pomegranate peel	SRX034876	159,871
	Musa cavendish	SRX146849	169,992

Supplementary Table 8. Tissue-specific small RNA-seq reads mapping in rice plastome.

Tissues	Normalized reads*	Mapped reads	Coverage (%)
**Root**	15,004,156	1,757 (0.01%)	6.3
**PABF**	15,004,156	11,436 (0.07%)	25.0
**Leaf**	15,004,156	420,375 (2.80%)	91.5
**Shoot**	15,004,156	574,317 (3.82%)	94.2

*All reads were normalized to contain same number of reads by randomly extraction from different tissues.

REFERENCES

43. T. Lu *et al*., Function annotation of the rice transcriptome at single-nucleotide resolution by RNA-seq. *Genome Research* **20**, 1238-1249 (2010).

44. Y. Marquez, J. W. Brown, C. Simpson, A. Barta, M. Kalyna, Transcriptome survey reveals increased complexity of the alternative splicing landscape in *Arabidopsis*. *Genome Research* **22**, 1184-1195 (2012).

45. R. Lister *et al*., Highly integrated single-base resolution maps of the epigenome in *Arabidopsis*. *Cell* **133**, 523-536 (2008).

46. D. C. Price *et al*., *Cyanophora paradoxa* genome elucidates origin of photosynthesis in algae and plants. *Science* **335**, 843-847 (2012).
